# Hair cortisol and self-perceived stress in adolescents with multi-system functional somatic disorders

**DOI:** 10.1186/s12888-024-05518-4

**Published:** 2024-02-05

**Authors:** Rebecca Nyengaard, Karen Hansen Kallesøe, Martin Køster Rimvall, Eva Ørnbøl, Kaare Bro Wellnitz, Else Marie Olsen, Vegard Bruun Bratholm Wyller, Charlotte Ulrikka Rask

**Affiliations:** 1 Research Unit Department of Child and Adolescent Psychiatry, Psychiatry, Aarhus University Hospital, Palle Juul-Jensens Boulevard 175, Aarhus N, 8200 Denmark; 2 Department of Clinical Medicine, Aarhus University, Incuba Skejby, building 2, Palle Juul-Jensens Boulevard 82, 8200 Aarhus N, Denmark; 3 Child and Adolescent Mental Health Centre, Mental Health Services in the Capital Region of Denmark, Kildegaardsvej 28, Entrance 3A, 1st Floor, 2900 Hellerup, Denmark; 4 Department of Child and Adolescent Psychiatry, Psychiatry Region Zealand, Ny Østergade 12, 4000 Roskilde, Denmark; 5 Research Clinic for Functional Disorders and Psychosomatics, Aarhus University Hospital, Palle Juul-Jensens Boulevard 11, Aarhus N, 8200 Denmark; 6 Centre for Clinical Research and Prevention, Bispebjerg and Frederiksberg Hospital, Ndr. Fasanvej 57, 1st Floor, Building 14, 2000 Frederiksberg, Denmark; 7 Psychiatric Centre Ballerup, Mental Health Services in the Capital Region of Denmark, Maglevænget 2, 2750 Ballerup, Denmark; 8 Department of Pediatric and Adolescent Medicine, Akershus University Hospital, Postboks 1000, 1478 Lørenskog, Norway; 9 Institute of Clinical Medicine, University of Oslo, Blindern, P.O box 1171, 0318 Oslo, Norway

**Keywords:** Adolescent, Chronic stress, Functional somatic disorder, Hair cortisol, Hypothalamic–pituitary–adrenal axis, Perceived stress

## Abstract

**Background:**

Long-term stress causing altered hypothalamic–pituitary–adrenal (HPA) axis dynamics with cortisol dysfunction may be involved in the pathophysiology of functional somatic disorders (FSD), but studies on adolescents with multi-system FSD are lacking. Therefore, we investigated: 1) whether hair cortisol concentration (HCC) differentiates adolescents with multi-system FSD from a) a population-based sample and b) a subgroup derived from the sample reporting a high physical symptom load, and 2) whether FSD population HCC is associated with primary symptom presentations and self-perceived stress.

**Methods:**

We used data from a clinical sample with multi-system FSD (*N* = 91, age 15–19 years) and a population-based sample (*N* = 1,450, age 16–17 years) including a subgroup with top 10% total scores on physical symptoms (*N* = 147). Density plots and multiple linear regression were applied to compare HCC between groups. In the clinical sample, multiple linear regression was employed to assess the association between HCC and primary symptom clusters and self-perceived stress.

**Results:**

Median HCC was lower in the clinical sample than in the population-based sample (β = 0.80 (95%CI: 0.66, 0.97)), but not significantly different from median HCC in the derived subgroup (β = 0.84 (95%CI: 0.66, 1.07)). In the clinical sample, HCC was not significantly associated with primary symptom clusters (F(2, 82) = 0.13, *p* = 0.88) or self-perceived stress (F(4, 83) = 1.18, *p* = 0.33).

**Conclusion:**

Our findings indicate that HCC is lowered in adolescents with multi-system FSD but not significantly associated with primary symptom presentations or self-perceived stress. Future studies including multiple measures of HPA axis dynamics alongside psychological measures may further elucidate the role of long-term stress in FSD.

**Trial registration:**

The AHEAD study was pre-registered at ClinicalTrials.gov (NCT02346071), 26/01/2015

**Supplementary Information:**

The online version contains supplementary material available at 10.1186/s12888-024-05518-4.

## Introduction

Functional somatic disorders (FSD) are increasingly common in adolescents with current prevalence estimates falling in the 4–10% range [1–3]. FSD are characterized by persistent physical symptoms that cannot be attributed to well-defined somatic disorders, leading to impairment, distress and high healthcare use [4–8]. FSD symptoms have been shown to broadly cluster by organ systems, also in young people; and evidence suggests that multi-symptom presentations predict severity and poorer long-term prognosis [9–12]. Accordingly, a recently proposed classification system divides FSD into single-symptom, single-system or multi-system FSD depending on the number of symptoms and symptom clusters involved [7].

To prevent the detrimental consequences of FSD, a better understanding of their underlying pathogenesis is needed. Currently, a complex interaction between biological, psychological and environmental factors is assumed, and adult studies have suggested that long-term stress with altered hypothalamic–pituitary–adrenal (HPA) axis dynamics may play a central role [7, 8, 13–15]. In general, acute exposure to stressors is known to initially cause elevated serum cortisol levels (hypercortisolism), whereas long-standing or chronic exposure may lead to an attenuation of the HPA axis with a dysregulated hormone secretion pattern and lowered cortisol levels (hypocortisolism) [16, 17]. It has therefore been hypothesized that hyper- or hypocortisolism – depending on stress duration – may be common in patients with FSD. However, extant studies on children or adolescents with single-symptom or single-system FSD have exclusively applied short-term cortisol measures. Such measures are strongly influenced by daily cortisol fluctuations [18]. Therefore, current findings from young populations are strikingly inconsistent, showing normo-, hypo- and hypercortisolism both within and between different FSD subtypes [19–26]. In contrast, a synthesis of results from multiple adult studies applying short-term cortisol measures found more specific evidence of hypocortisolism in chronic fatigue syndrome and in fibromyalgia, but normocortisolism in irritable bowel syndrome [15]. This indicates that HPA axis alterations may differ between FSD subtypes.

In sum, studies investigating long-term cortisol levels in young people with FSD are highly needed. For this purpose, hair cortisol concentration (HCC) is a promising measure. HCC alterations have been found in both acutely and chronically stressed individuals. However, characteristics such as sex, age and anthropometric measures may also influence HCC levels [27, 28]. Currently, the literature on HCC in FSD populations is limited to a few adult studies which primarily found unaltered or lowered HCC [29–32]. Thus, these results are partly in accordance with findings from adult studies applying short-term cortisol measures.

To the best of our knowledge, no previous studies have examined HCC in youths diagnosed with multi-system FSD with various symptom presentations. The present study aimed to bridge this gap by:

1. comparing HCC between a clinical sample of adolescents with multi-system FSD and a) a population-based sample of adolescents and b) among these a subgroup reporting a high physical symptom load, in order to validate potential specific HCC findings for the clinical sample.
2. investigating the association within the clinical sample between HCC and a) clusters of primary symptoms, i.e. cardiopulmonary/autonomic, gastrointestinal, musculoskeletal and general symptoms (including fatigue) and b) self-perceived stress.

We hypothesized that the majority of the clinical sample would display lowered HCC because of long-lasting stress experiences and that a minor fraction would display elevated HCC because of more short-lived, acute stress experiences; that HCC in the subgroup with high physical symptom load derived from the population-based sample would resemble HCC in the clinical sample; that HCC in the clinical sample would be particularly lowered in those reporting primary symptoms from the general and musculoskeletal symptom clusters; and that high self-perceived stress would be associated with both low and high HCC because of stress-induced hypo- or hypercortisolism.

## Methods

### Study populations

The samples were derived from two Danish studies: a) the Acceptance and Commitment Therapy for Health in Adolescents (AHEAD) study [33, 34] and b) the 16–17-year follow-up of the Copenhagen Child Cohort 2000 (CCC2000) [35].

The AHEAD study was a randomized controlled trial conducted from January 2015 to November 2019. The trial compared group-based psychological treatment (i.e. Acceptance and Commitment Therapy (ACT)) with enhanced usual care and included a clinical sample of 15–19-year-old adolescents (*N* = 91) diagnosed with multi-system FSD conceptualised as multi-organ bodily distress syndrome (BDS) with a minimum duration of 12 months [36, 37]. To meet the diagnostic criteria of multi-organ BDS, the participants needed to report a) symptoms from at least three of four symptom clusters, i.e. cardiopulmonary/autonomic, gastrointestinal, musculoskeletal and general symptoms; b) at least three symptoms from at least three clusters; and c) moderate to severe impairment in daily life.

The CCC2000 is a general population-based birth cohort including all 6,090 children born in a specified suburban area of Copenhagen in year 2000. At baseline, the cohort was generally representative of the Danish child population [35]. Herein, we used data from 1,450 cohort members who donated hair samples during the 16–17-year follow-up between August 2016 and November 2017. Among these, 147 participants constituted a subgroup with a high physical symptom load, reporting top 10% total scores on the BDS checklist (see Measures section). This cut-off was used in a previous comparison study between AHEAD and CCC2000 [38]. At the 16–17-year follow-up lower participation rates were seen among cohort members from families of lower socioeconomic status, with an immigrant background and with high familial loads of psychopathology [35]. In both samples, participants were included throughout the course of the year.

### Procedures

The procedures have been described in detail elsewhere [33, 35].

In short, AHEAD participants underwent a thorough clinical assessment by trained physicians, including a medical record review, a standardized clinical interview, the Schedules for Clinical Assessment in Neuropsychiatry (SCAN) interview [39], a clinician performed screening for potential developmental disorders based on diagnostic criteria, a clinical neurological examination and standard blood tests. Hair samples for HCC analysis and responses to web-based questionnaires were obtained at baseline before intervention.

Eligible CCC2000 members were notified of the 16–17-year follow-up through the Danish national online mailbox system. Following this, participants answered web-based questionnaires and hair samples were obtained at face-to-face examinations.

Data from both studies were pseudonymized before being uploaded to and analysed on the servers of Statistics Denmark, a secure remote access system [40].

### Measures

#### Hair cortisol concentration (HCC)

Hair sampling procedures were identical in AHEAD and CCC2000. Two strands of hair, each measuring approximately 3 mm in diameter, were cut from each participant from the posterior vertex position right by the scalp. The scalp-near ends were marked, and samples were stored in aluminium foil. Samples were analysed at the laboratory of the Chair of Biopsychology, Technische Universität Dresden, as a single batch (January 2020) using 7.5 mg of 2 cm long non-pulverised scalp-near hair strands. As the average hair growth rate is reported to be 1 cm/month [41], these samples contained the amount of cortisol secreted during the two preceding months. Hair sample wash and steroid extraction procedure followed the laboratory protocol [42]. HCC was determined using immunoassays with chemiluminescence detection (CLIA, IBL International, Hamburg, Germany). The intra- and inter-assay coefficients of variance of this assay are below 8% [42].

#### Self-perceived stress

In AHEAD, self-perceived stress was measured using a validated Danish version of the ten-item Perceived Stress Scale (PSS-10), a widely used self-report instrument assessing an individual's perceived stress during the preceding month [43, 44]. The items are rated on a five-point Likert scale (total score: 0–40) with higher scores indicating higher degrees of self-perceived stress. The scale has demonstrated acceptable psychometric properties in adult populations [45], and Cronbach's α was 0.91 in AHEAD.

#### Functional somatic symptoms

In both samples, physical symptoms were assessed by the 25-item BDS checklist, a validated self-report questionnaire developed to identify individuals with probable FSD by assessing physical symptoms from four symptom clusters: cardiopulmonary/autonomic, gastrointestinal, musculoskeletal and general symptoms [11, 46, 47]. The items are rated on a five-point Likert scale (total score: 0–100) with higher scores indicating a higher symptom load. The time frame for symptom registration was four weeks in AHEAD and one year in CCC2000. Cronbach's α was 0.87 in AHEAD and 0.90 in CCC2000. The questionnaire has recently been validated in two general population-based studies including participants down to 14 years old [12, 47].

The SCAN interview in AHEAD contained a detailed section on functional somatic symptoms, including an assessment of each patient's primary functional somatic symptom. For the purpose of the present study, primary symptoms were categorised into the four symptom clusters described above. A few (n ≤ 5) patients reported pseudo-neurological primary symptoms not included in the pre-defined symptom clusters such as visual disturbances and were placed in the general or musculoskeletal clusters based on expert consensus.

#### Sociodemographic and anthropometric data

Information on sex and age was based on self-reported information in AHEAD and on register data in CCC2000 [48]. In both studies, weight and height were measured as part of face-to-face physical examinations.

#### Additional characteristics for descriptive purposes

Psychiatric and somatic morbidity and medication use were registered as part of the baseline clinical assessment in AHEAD. In CCC2000, hospital-registered lifetime International Classification of Diseases, tenth version (ICD-10) diagnoses from birth to end of follow-up in June 2017 were obtained through the Danish National Patient Register [48, 49]. Information on physician-diagnosed chronic somatic disorders such as asthma or diabetes was obtained through self-report items from the Soma Assessment Interview (SAI) [50]. Furthermore, information on parental cohabitation and parental education level was based on parent-reported data in AHEAD and on register data in CCC2000.

### Statistical analyses

Descriptive data were reported using means (standard deviation [SD]), medians (interquartile range [IQR]) or frequencies (%).

To fulfil aim 1, density plots were created to visually compare HCC distributions between the AHEAD sample and a) the total CCC2000 sample and b) the CCC2000 subgroup with a high symptom load. Multiple linear regression was used to assess the association between HCC (log-transformed) and study sample, adjusting for sex, age and Body Mass Index (BMI, log-transformed). The confounding effect of these factors has been confirmed in multiple studies [27, 28]. Further variables were not included in the analyses due to sample size limitations and due to use of different measurement methods in AHEAD and CCC2000. Two sensitivity analyses were performed: a) excluding AHEAD participants using systemic glucocorticoid medication and CCC2000 participants reporting physician-diagnosed chronic somatic disorders and b) excluding potentially invalid HCC measurements, i.e. due to insufficient (≤ 5 mg) or lacking hair sample weight, incorrect or lacking information about hair sampling position and extremely high HCC values (> 99 percentile of the CCC2000 sample).

To fulfil aim 2, multiple linear regression was performed on AHEAD data to assess the association between a) HCC (log-transformed) and primary symptom clusters and b) self-perceived stress total score and HCC. For the latter, HCC was modelled using restricted cubic splines to allow for non-linearity because of the expected curvilinear relationship. Sex, age and BMI were included as covariates. Possible influential outliers were detected using leverages and Cook's D and were excluded in the sensitivity analyses.

In a supplementary analysis, Pearson's correlation coefficient was calculated to investigate the correlation between illness duration (log-transformed) and HCC (log-transformed) in AHEAD.

Throughout, diagnostic plots were prepared to explore assumptions of linearity, homoscedasticity and normality of residuals. Results are reported with 95% confidence intervals (CI). Stata version 17.0 was used for the analyses [51].

Power analyses were not performed due to the secondary nature of the present study.

### Ethics

The study was conducted in accordance with the Declaration of Helsinki (2013). The AHEAD study was approved by the Committee on Health Research Ethics of the Central Denmark Region (1–10-72–181-14) and the Danish Data Protection Agency (1–16-02–290-14) and pre-registered with ClinicalTrials.gov (NCT02346071). The CCC2000 study was approved by the Danish Data Protection Agency (CSU-FCFS-2016–004, I-Suite 04544) and the Local Committee on Health Research Ethics (protocol 16,023,242), and updated in PACTIUS (the data system of the Capital Region) in 2021 (P-2021–720). Written informed consent was obtained from all participants and if age < 18 years their legal guardians.

## Results

### Participant characteristics

Table 1 displays participant characteristics for each sample. AHEAD and the CCC2000 subgroup with a high symptom load had similar mean BDS checklist total scores and a higher prevalence of psychiatric disorders. Their sex distributions were also similar; the majority were girls, whereas only about half were girls in the total CCC2000 sample. In AHEAD, the median age was higher and the age variance was wider than in CCC2000, and the median duration of FSD was 3.4 years (IQR: 2.33–5.17). To investigate whether illness duration correlated with HCC in AHEAD, Pearson's correlation analysis was performed. This analysis showed that log-transformed HCC did not correlate with log-transformed FSD duration in months: r(89) = -0.04 (95%CI: -0.24, 0.17).

**Table 1:** Participant characteristics

	**AHEAD**	**CCC2000** **(total sample)**	**CCC2000 subgroup**^**a**^ **(high physical symptom load)**
	**(*n* = 91)**	**(*n* = 1,450)**	**(*n* = 147)**
**Sex (female: n (%))**	82 (90.1)	827 (57.0)	120 (81.6)
**Age (years: median (IQR))**	17.9 (16.6–19.4)	16.6 (16.4–16.8)	16.6 (16.4–16.8)
**BMI (mean (SD))**	22.3 (3.6)	21.8 (3.6)	22.7 (3.9)
**Psychiatric diagnoses (n (%))**^**b**^			
1.Neurodevelopmental	3 (3.3)	91 (6.3)	20 (13.6)
2.Emotional	40 (44.0)	84 (5.8)	29 (19.7)
3.Other	n < 3	44 (3.0)	11 (7.5)
Any	40 (44.0)	136 (9.4)	34 (23.1)
**BDS checklist (score 0–100)**^**c**^			
Mean (SD)	44.4 (15.9)	16.5 (12.4)	45.9 (9.7)
Median (IQR)	43.0 (32.0–56.0)	13.0 (8.0–22.0)	42.5 (38.0–52.0)
**Primary symptom cluster**^**d**^** (n (%))**			
1.General symptom cluster	52 (57.1)	-	-
2.Musculoskeletal symptom cluster	17 (18.7)	-	-
3.Gastrointestinal symptom cluster	19 (20.9)	-	-
4.Cardiopulmonary/autonomic symptom cluster	3 (3.3)	-	-
**Parental cohabitation (living together: n (%))**	56 (61.5)	1070 (73.8)^e^	109 (74.2)^f^
**Father's highest level of education (n (%))**			
1.Short (high school or below)	27 (29.7)	256 (17.7)	35 (23.8)
2.Medium (vocational, bachelor or equivalent)	39 (42.9)	893 (61.6)	86 (58.5)
3.Higher (master or equivalent)	16 (17.6)	252 (17.4)	16 (10.9)
*Missing*	*9 (9.9)*	*49 (3.4)*	*10 (6.8)*
**Mother's highest level of education (n (%))**			
1.Short (high school or below)	26 (28.6)	245 (16.9)	34 (23.1)^g^
2.Medium (vocational, bachelor or equivalent)	52 (57.1)	963 (66.4)	97 (66.0)
3.Higher (master or equivalent)	8 (8.8)	233 (16.1)	16 (10.9)
*Missing*	*5 (5.5)*	*9 (0.6)*	*(See *^*g*^*)*

*AHEAD* Acceptance and Commitment Therapy for Health in Adolescents, *BDS* bodily distress syndrome, *BMI* Body Mass Index, *CCC2000* Copenhagen Child Cohort 2000, *IQR* interquartile range, *n* number in specific population, *SD* standard deviation

^a^The CCC2000 subgroup consists of the CCC2000 participants reporting within the 10% highest scores on the BDS checklist

^b^In both samples, psychiatric disorders were categorized into three groups: neurodevelopmental (hyperactivity and inattention disorders, conduct disorders, autism spectrum disorders and intellectual disability), emotional (depressive disorders and anxiety disorders), other (substance abuse-related disorders, psychotic disorders, eating disorders and personality disorders) [48]. Selected psychiatric disorders, including severe neurodevelopmental disorders, psychotic disorders and substance abuse, were part of the AHEAD exclusion criteria [33]

^c^The time frame for symptom reporting was 4 weeks in AHEAD, and one year in CCC2000

^d^The patients' primary symptom presentations were categorized according to the four BDS symptom clusters

^e^Information on parental cohabitation was missing in 18 (1.25%) of the CCC2000 participants. Among these, 14 had moved out of their parents' home

^f^Information on parental cohabitation was missing in three (2.04%) of the adolescents in the CCC2000 subgroup. All had moved out of their parents' home

^g^Due to few (n ≤ 3) observations in the 'Missing' group, these were included in the group of mothers with 'Short' educations

### Comparison of hair cortisol concentrations between samples

**Fig. 1:**
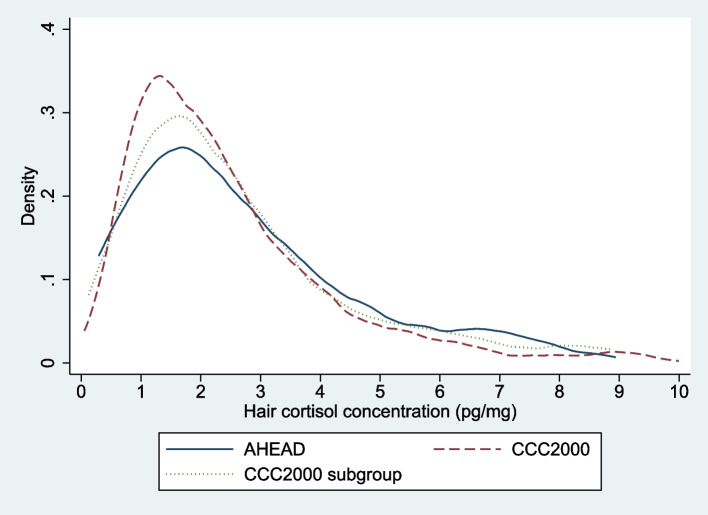
Crude hair cortisol concentration distributions in AHEAD (*n* = 91) and CCC2000 (total sample (*n* = 1,450) and subgroup with a high physical symptom load (*n* = 147)) *AHEAD* Acceptance and Commitment Therapy for Health in Adolescents, *CCC2000* Copenhagen Child Cohort 2000, *pg/mg* picograms/milligram. The figure shows the crude density plot for each sample. The crude medians were 2.2 (95%CI: 1.8, 2.7; IQR = 1.2-3.8) pg/mg in AHEAD, 2.1 (95%CI: 1.9, 2.1; IQR = 1.2-3.3) pg/mg in CCC2000 and 2.2 (95%CI: 1.9, 2.6; IQR = 1.3-3.5) pg/mg in the CCC2000 subgroup. The layout of the density plots has been modified to ensure that individual observations are unidentifiable. In accordance with this, high HCC values have been removed: for AHEAD and the CCC2000 subgroup, only HCC values between 0.0-9.0 pg/mg are shown (excluding ≤ 5 participants from each sample); for CCC2000, only HCC values between 0.0-10.0 pg/mg are shown (excluding 37 participants)

Figure 1 shows density plots of the crude HCC distributions in AHEAD and CCC2000 (total sample and subgroup). All distributions were similar as they were generally unimodal, positively skewed and had similar medians.

Results from multiple linear regression analyses with comparisons of HCC between AHEAD and CCC2000 (total sample and subgroup, respectively) are shown in Table 2.

**Table 2:** Comparison of hair cortisol concentrations between AHEAD and CCC2000 (whole sample and subgroup with a high physical symptom load) using multiple linear regression

Model	Dependent variable: HCC^a^		
	**β**	**95%CI**	**P**
**CCC2000 compared with AHEAD**			
**Crude**			
CCC2000 (*n* = 1,450)	1 (ref.)		
AHEAD (*n* = 91)	1.05	0.88; 1.24	0.590
**Adjusted for sex, age and BMI**^**a**^			
CCC2000 (*n* = ^b^)	1 (ref.)		
AHEAD^c^ (*n* = 91)	0.80	0.66; 0.97	0.023*
**CCC2000 subgroup with a high physical symptom load compared with AHEAD**			
**Crude**			
CCC2000 subgroup (*n* = 147)	1 (ref.)		
AHEAD (*n* = 91)	0.97	0.79; 1.20	0.786
**Adjusted for sex, age and BMI**^**a**^			
CCC2000 subgroup (*n* = 147)	1 (ref.)		
AHEAD^d^ (*n* = 91)	0.84	0.66; 1.07	0.156

*AHEAD* Acceptance and Commitment Therapy for Health in Adolescents, *BMI* Body Mass Index, *CCC2000* Copenhagen Child Cohort 2000, *CI* confidence interval, *HCC* hair cortisol concentration, *n* number in specific population, *ref*. reference group

^a^HCC and BMI were log-transformed for analyses; the table presents the back-transformed values

^b^Due to missing covariate data in CCC2000, ≤ 5 persons were excluded in the adjusted analysis

^c^Interpretation (adjusted analysis comparing the CCC2000 total sample to AHEAD): if two persons from CCC2000 and AHEAD with the same sex, age and BMI are compared with each other, the person from AHEAD will have a 20% (95%CI: 3%, 34%) lower median HCC than the person from CCC2000. For example, the median HCC in a 17-year-old girl from AHEAD with BMI 21 will be 1.9 (95%CI: 1.6, 2.2) pg/mg, whereas the median HCC in an equivalent participant from CCC2000 will be 2.3 (95%CI: 2.2, 2.5) pg/mg

^d^Interpretation (adjusted analysis comparing the CCC2000 subgroup to AHEAD): if two persons from the CCC2000 subgroup and AHEAD with the same sex, age and BMI are compared with each other, the person from AHEAD will have a 16% (95%CI: -7%, 34%) lower median HCC than the person from the CCC2000 subgroup

^*^ p < 0.05

No significant differences were found in the crude models. In the adjusted models, the median HCC in AHEAD was 20% (95%CI: 3%, 34%) lower than in the total CCC2000 sample, whereas the ratio of HCC medians remained statistically non-significantly different between AHEAD and the CCC2000 subgroup.

In sensitivity analyses excluding a) AHEAD participants using glucocorticoid medication and participants from the total CCC2000 total sample with chronic somatic disorders and b) potentially invalid HCC measurements, results became statistically non-significant (see Supplementary Table ST1 and ST2). However, all adjusted models showed trends towards HCC medians in AHEAD being lower than in both the total CCC2000 sample and the CCC2000 subgroup.

### Comparison of hair cortisol concentrations between primary symptom clusters in AHEAD

Table 3 presents results from multiple linear regression-based comparisons of HCC between primary symptom clusters in AHEAD.

**Table 3:** Comparison of hair cortisol concentrations between primary symptom clusters in AHEAD using multiple linear regression

Model^b^	Dependent variable: HCC^a^		
	**β**	**95%CI**	**P**
**Crude**			
General symptom cluster (*n* = 52)	1 (ref.)		
Musculoskeletal symptom cluster (*n* = 17)	0.90	0.56; 1.42	0.635
Gastrointestinal symptom cluster (*n* = 19)	1.06	0.68; 1.64	0.809
**Adjusted for sex, age and BMI**			
General symptom cluster (*n* = 52)	1 (ref.)		
Musculoskeletal symptom cluster^c^ (*n* = 17)	0.93	0.58; 1.48	0.744
Gastrointestinal symptom cluster^d^ (*n* = 19)	1.07	0.69; 1.66	0.775

*BMI* Body Mass Index, *CI* confidence interval, *HCC* hair cortisol concentration, *n* number in specific population, *ref*. reference group

^a^HCC was log-transformed for analyses; the table contains the back-transformed values

^b^Due to a low number of patients (*n* = 3) in the cardiopulmonary/autonomic primary symptom cluster, data from this cluster were not included in the analyses

^c^Interpretation (adjusted analysis comparing participants with primary symptoms from the general symptom cluster to participants with primary symptoms from the musculoskeletal symptom cluster): if two participants belonging to the general or musculoskeletal primary symptom cluster with the same sex, age and BMI are compared with each other, the participant with a musculoskeletal primary symptom will have a 7% (95%CI: -48%, 42%) lower median HCC than the participant with a general primary symptom

^d^Interpretation (adjusted analysis comparing participants with primary symptoms from the general symptom cluster to participants with primary symptoms from the gastrointestinal symptom cluster): if two participants belonging to the general or gastrointestinal primary symptom cluster with the same sex, age and BMI are compared with each other, the participant with a gastrointestinal primary symptom will have a 7% (95%CI: -31%, 66%) higher median HCC than the participant with a general primary symptom

Based on the crude model, median HCC values in the participants from the three largest primary symptom clusters were: general symptom cluster: 2.2 (95%CI: 1.8, 2.8) pg/mg, musculoskeletal symptom cluster: 2.0 (95%CI: 1.3, 3.0) pg/mg, gastrointestinal symptom cluster: 2.3 (95%CI: 1.6, 3.4) pg/mg. Primary symptom clusters were not significantly associated with HCC in the crude (F(2, 85) = 0.18, *p *= 0.83) or the adjusted (F(2, 82) = 0.13, *p* = 0.88) analyses.

### Association between hair cortisol concentration and self-perceived stress in AHEAD

**Fig. 2:**
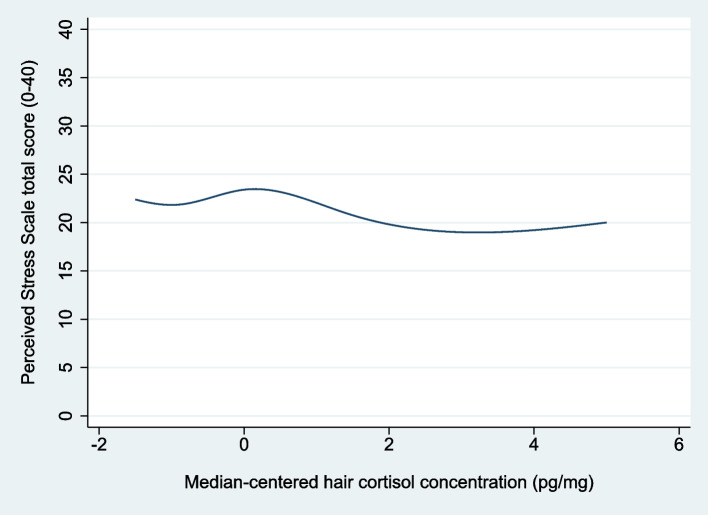
Crude association between hair cortisol concentration and self-perceived stress in AHEAD *AHEAD* Acceptance and Commitment Therapy for Health in Adolescents, *HCC* hair cortisol concentration, *pg/mg *picograms/milligram. The figure shows the plot of the crude linear regression model. HCC was centered at the median (2.2 pg/mg) for analyses (i.e. median-centered HCC = original value -2.2 pg/mg). The layout of the graph has been modified to ensure that individual observations are unidentifiable. In accordance with this, data from the five patients with the lowest HCC and the five patients with the highest HCC have been removed

In AHEAD, the mean PSS total score was 22.05 (SD: 8.60) and the median time interval between responding to the PSS questionnaire and hair sampling was 9 (IQR: 3–16) days. Figure 2 presents a plot of the crude regression model examining the association between HCC and PSS total score.

HCC was not significantly associated with PSS total score, i.e. HCC splines were not significantly different from 0 in the crude (F(4, 86) = 1.19, *p* = 0.32) or in the adjusted (F(4, 83) = 1.18, *p* = 0.33) analyses (see detailed results in Supplementary Table ST3). Possible influential outliers (n ≤ 3) were excluded in the sensitivity analyses, leaving the overall results unchanged (results not shown).

## Discussion

### Main findings

This study showed that HCC was significantly lower in a clinical sample of adolescents with severe, multi-system FSD than in a general population-based sample of adolescents when adjusting for sex, age and BMI but not lower than HCC in a subgroup reporting a high physical symptom load. Within the clinical sample, HCC did not differ significantly between different primary symptom presentations, and HCC was not significantly associated with self-perceived stress.

### Strengths and limitations

The main strengths of this study include the use of data on adolescents with multi-system FSD who underwent thorough clinical assessment, were diagnosed in accordance with empirically-validated diagnostic criteria and were compared with a large general population-based sample. Moreover, the hair sampling procedures were identical, and all hair samples were analysed together in one batch, thereby limiting the risk of bias due to measurement errors. Even so, the results should be interpreted in light of several limitations. First, due to the cross-sectional design, the temporality and causality underlying our findings could not be investigated. Second, only one measure of HPA axis activity (i.e. HCC) and only one measure of self-perceived stress (i.e. the PSS) were included although these measures might not adequately reflect the complex nature of the HPA axis and psychological stress phenomena. In addition, HCC may have been confounded by factors for which we could not duly adjust due to use of different measurement methods and sample size limitations, including hair characteristics, psychiatric illnesses and medication use. However, current literature regarding the influence of these factors on HCC is conflicting. Therefore, we included only sex, age and BMI as covariates – the effect of which has been confirmed in various studies [27, 28]. Third, the difference in HCC between AHEAD and CCC2000 was attenuated in the sensitivity analyses where the results became statistically non-significant. However, across all confounder-adjusted analyses, we found a consistent trend towards HCC in AHEAD being lower than in the CCC2000 total sample. Fourth, the sample size of AHEAD may have limited the power of the restricted cubic splines analysis pertaining to our hypothesis of curvilinear HCC, where a subsample with elevated HCC may have been too small to detect. Still, despite a limited sample size of AHEAD, we did partially confirm our hypothesis with an overall finding of hypocortisolism when comparing the AHEAD sample and the CCC2000 sample. Fifth, the study samples were drawn from two different studies with different recruitment procedures. Thus, the clinical sample in AHEAD consisted of patients referred to a treatment study, whereas the CCC2000 sample consisted of those members of a general population-based birth cohort who consented to donate hair samples for a follow-up study. Therefore, the clinical sample may have consisted of the most ill fraction of the eligible participants, whereas the CCC2000 follow-up study consisted of the most resourceful cohort members. This may have led to selection bias and overestimation of the difference in HCC between the samples. However, while no exclusion criteria were applied in CCC2000, the exclusion criteria in AHEAD may have led to exclusion of some of the most ill and least resourceful patients (e.g. patients with mental retardation, psychotic disorders and substance abuse). Moreover, the intensive treatment programme and long-distance travelling may also have prohibited some less resourceful patients from participating. Therefore, the resulting degree of selection bias may have been limited.

### Comparison with previous studies

The finding of lower HCC in adolescents with multi-system FSD concurs with two previous studies investigating HCC levels in adults with functional somatic syndromes [29, 30]. The largest of these included 169 patients with irritable bowel syndrome and 316 sex- and age-matched controls. This study found lower HCC in patients, corroborating our findings. On the other hand, two smaller adult case–control studies found largely identical HCC in patients and healthy controls, and a small case–control study even found higher HCC in patients with chronic pain [31, 32, 52]. However, the latter patient sample differed from typical FSD populations by primarily experiencing pain caused by well-defined chronic somatic disorders such as lumbar degenerative disc disease. Moreover, all patients received long-term opioid treatment. Nevertheless, we hypothesized that a small fraction of our clinical sample would display elevated HCC due to short-term stress. However, the HCC distribution in our clinical sample appeared to be unimodal, which may reflect that the AHEAD sample was too small to detect minor subgroups. Conversely, it could be speculated that all the patients in the clinical sample were hypocortisolemic owing to long-term stress caused by their long-lasting illness.

Taken together, our findings may provide support for the theory that long-term stress with HPA axis attenuation and resulting hypocortisolism are involved in the pathophysiology of multi-system FSD; even so, studies investigating long-term cortisol levels in patients with multi-system FSD for comparison are generally lacking [25]. However, a few studies applying short-term cortisol measures do exist, including a recent case–control study on 151 adult mainly female patients with multi-system FSD. This study found reduced levels of serum cortisol and a negative correlation between cumulative chronic stress scores and serum cortisol in females, thus providing preliminary support for the involvement of chronic stress and hypocortisolism in multi-system FSD [53]. Alternatively, the heterogeneous results of existing studies investigating HPA axis dynamics in FSD could suggest that the primary phenomenon involves a dysregulated pattern of hormone secretion, resulting in an attenuated diurnal variation of cortisol and a reduced responsiveness of the HPA axis but limited deviations in long-term cortisol levels as reflected in HCC [13, 15, 17]. Supporting this theory, studies on both adolescent and adult FSD populations with single-system presentations have found altered patterns of daily cortisol levels and altered responses to hormone stimulation tests [21, 24, 54].

As hypothesized, no statistically significant differences were found between HCC in AHEAD and the CCC2000 subgroup with a high physical symptom load. As cortisol is a key hormone in most endocrine signalling pathways, it is expected that the biology underlying unspecific physical symptoms arising on the basis of distress is the same. However, a trend was observed towards HCC being lower in the clinical sample across all confounder-adjusted analyses, including subgroup analyses. Thus, although the BDS checklist scores were similar in these two groups, it is likely that our results reflect that the clinical sample, being referred to hospital-based care, represented a more impaired group with long-lasting FSD. This may, again, potentially indicate a longer stress duration leading to more substantial HPA axis attenuation and hypocortisolism. In addition, it could be speculated that the BDS checklist symptom scores in CCC2000 were high because of the long time frame for symptom registration (i.e. one year) in this sample. Finally, our finding of no correlation between HCC and illness duration in the clinical sample may indicate that long-standing illness had caused most of the patients to "switch" to a static, chronic state of hypocortisolism that no longer correlated with the duration of their illness and their experience of stress.

The finding that HCC in adolescents with FSD did not differ significantly according to primary symptom presentations may potentially indicate that pathophysiological alterations largely overlap between FSD subtypes. However, the lack of differences could also be explained by the fact that all patients exhibited symptoms from multiple symptom clusters, making any subdivision based on primary symptoms imprecise. Furthermore, the low number of patients in each cluster reduced the statistical power. Thus, HCC differences may exist between more clear-cut FSD subtypes or earlier in the course of FSD when single-symptom or single-system presentations are more common. Currently, results from studies applying short-term cortisol measures offer preliminary support for this theory. Accordingly, a meta-analysis of adult studies applying short-term measures found evidence of hypocortisolism only in chronic fatigue syndrome and in females with fibromyalgia. Moreover, a study of adolescents found a symptom cluster comprising headache and gastrointestinal symptoms to be associated with low cortisol during a stress task; and another cluster comprising overtiredness, dizziness and musculoskeletal pain to be associated with low cortisol after awakening [15, 26].

Finally, the finding that HCC was not significantly associated with self-perceived stress in adolescents with FSD is in line with most existing literature, including studies on adolescents and young adults [27, 55–59]. This indicates that self-perceived stress may not correlate with the physiological stress response, possibly due to individual differences in awareness of stress experiences or a limited sensitivity of existing self-perceived stress measures [60]. The former could especially be the case for young patients with FSD due to a potentially higher level of alexithymia with difficulties recognizing and describing inner experiences [61]. Still, the mean PSS total score of 22.05 in the present study was higher than in two large general population-based samples from Germany (including participants ≥ 14 years old) and Sweden recording mean total scores of 12.57 and 13.96, respectively [62, 63]. These results could suggest that our clinical sample did indeed experience overall higher subjective stress levels than those seen in the general population; a finding that also provides support for the involvement of stress in FSD.

## Conclusion

This study indicates that HCC is lower in adolescents with multi-system FSD than in adolescents from the general population, preliminarily supporting that attenuation of the HPA axis may be involved in the pathophysiology of FSD. The study does not provide evidence for differences in long-term cortisol levels between primary symptom clusters in FSD, or for an association between self-perceived stress and long-term cortisol levels in FSD. These findings underscore the need for larger samples in future studies incorporating both short- and long-term HPA axis measures and hormonal challenge tests alongside psychological stress measures to build an in-depth understanding of the role of long-term stress and HPA axis dynamics in the pathophysiology of FSD.

### Supplementary Information

**Additional file 1: Supplementary Table 1.** (ST1). **Supplementary Table 2.** (ST2). **Supplementary Table 3.** (ST3)..

## Data Availability

The data used in the present study contain sensitive personal information and therefore cannot be shared freely due to Danish data protection laws. All data (pseudonymised) are stored on a digital server at Statistics Denmark, and access can be granted through approval from the CCC2000 steering committee which has legal responsibility for data as the data manager. If access is granted, the principal investigator and last author (Charlotte Ulrikka Rask, charrask@rm.dk) will make data available through Statistics Denmark. The General Data Protection Regulation (GDPR) and the Danish Data Protection Act prohibit any other forms of data sharing.

## Abbreviations

AHEAD: Acceptance and Commitment Therapy for Health in Adolescents
BDS: Bodily Distress Syndrome
BMI: Body Mass Index
CCC2000: Copenhagen Child Cohort 2000
FSD: Functional somatic disorder
HCC: Hair cortisol concentration
HPA: Hypothalamic–pituitary–adrenal
PSS: Perceived Stress Scale
SAI: Soma Assessment Interview
SCAN: Schedules for Clinical Assessment in Neuropsychiatry
SD: Standard deviation
