# Supplementary material for: Hair cortisol and self-perceived stress in adolescents with multi-system functional somatic disorders
Source: BMC Psychiatry. 2024 Feb 5;24:101. doi: 10.1186/s12888-024-05518-4 (PMC10840144; doi:10.1186/s12888-024-05518-4)
Supplement: Supplementary file 1 — Additional file 1: Supplementary Table 1. (ST1). Supplementary Table 2. (ST2). Supplementary Table 3. (ST3). [file 12888_2024_5518_MOESM1_ESM.docx]

# Supplementary

## ***Supplementary Table 1 (ST1)***

## *Sensitivity analysis excluding AHEAD participants using glucocorticoid medication and CCC2000 participants with chronic somatic disorders using multiple linear regression*

|  | **Dependent variable: HCC^a^** | | |
| --- | --- | --- | --- |
| **Model** | **β** | **95%CI** | **P** |
| **CCC2000 compared with AHEAD** |  |  |  |
| **Crude** |  |  |  |
| - CCC2000 (n=1,102) | 1 (ref.) |  |  |
| - AHEAD (n=88) | 1.08 | 0.91; 1.28 | 0.357 |
| **Adjusted for sex, age and BMI^a^** |  |  |  |
| - CCC2000 (n=^b^) | 1 (ref.) |  |  |
| - AHEAD^c^ (n=88) | 0.85 | 0.69; 1.03 | 0.100 |
| **CCC2000 subgroup with a high physical symptom load compared with AHEAD** | | | |
| **Crude** |  |  |  |
| - CCC2000 subgroup (n=103) | 1 (ref.) |  |  |
| - AHEAD (n=88) | 0.98 | 0.79; 1.23 | 0.869 |
| **Adjusted for sex, age and BMI^a^** |  |  |  |
| - CCC2000 subgroup (n=103) | 1 (ref.) |  |  |
| - AHEAD^d^ (n=88) | 0.84 | 0.65; 1.09 | 0.190 |

*AHEAD: Acceptance and Commitment Therapy for Health in Adolescents, BMI: Body Mass Index, CCC2000: Copenhagen Child Cohort 2000, CI: confidence interval, HCC: hair cortisol concentration, n: number in specific population, ref.: reference group*

*^a^HCC and BMI were log-transformed for analyses; the table contains the back-transformed values.*

*^b^Due to missing covariate data in CCC2000, ≤5 persons were excluded in the adjusted analysis.*

*^c^Interpretation (adjusted analysis comparing participants from the CCC2000 total sample to participants from AHEAD): if two participants from CCC2000 or AHEAD with the same sex, age and BMI are compared with each other, the participant from AHEAD will have a 15% (95%CI: -3%, 31%) lower median HCC than the participant from CCC2000.*

*^d^Interpretation (adjusted analysis comparing participants from the CCC2000 subgroup to participants from AHEAD): if two persons from the CCC2000 subgroup or AHEAD with the same sex, age and BMI are compared with each other, the participant from AHEAD will have a 16% (95%CI: -9%, 35%) lower median HCC than the participant from the CCC2000 subgroup.*

## ***Supplementary Table 2 (ST2)***

## *Sensitivity analysis excluding potentially invalid hair cortisol concentration measurements^a^ using multiple linear regression*

|  | **Dependent variable: HCC^b^** | | |
| --- | --- | --- | --- |
| **Model** | **β** | **95%CI** | **P** |
| **CCC2000 compared with AHEAD** |  |  |  |
| **Crude** |  |  |  |
| - CCC2000 (n=1,077) | 1 (ref.) |  |  |
| - AHEAD (n=^c^) | 0.99 | 0.84; 1.16 | 0.905 |
| **Adjusted for sex, age and BMI^b^** |  |  |  |
| - CCC2000 (n=^d^) | 1 (ref.) |  |  |
| - AHEAD^e^ (n=^c^) | 0.84 | 0.69; 1.00 | 0.056 |
| **CCC2000 subgroup with a high physical symptom load compared with AHEAD** | | | |
| **Crude** |  |  |  |
| - CCC2000 subgroup (n=126) | 1 (ref.) |  |  |
| - AHEAD (n=^c^) | 0.93 | 0.75; 1.15 | 0.496 |
| **Adjusted for sex, age and BMI^b^** |  |  |  |
| - CCC2000 subgroup (n=^d^) | 1 (ref.) |  |  |
| - AHEAD^f^ (n=^c^) | 0.85 | 0.67; 1.08 | 0.175 |

*AHEAD: Acceptance and Commitment Therapy for Health in Adolescents, BMI: Body Mass Index, CCC2000: Copenhagen Child Cohort 2000, CI: confidence interval, HCC: hair cortisol concentration, n: number in specific population, ref.: reference group*

*^a^Samples with potentially invalid HCC measurements included samples with insufficient (≤ 5 mg) or lacking hair sample weight, incorrect or lacking information about hair sampling position, or extremely high HCC values (> 99 percentile of the CCC2000 total sample).*

*^b^HCC and BMI were log-transformed for analyses; the table contains the back-transformed values.*

*^c^Due to potentially invalid HCC measurements in AHEAD, ≤3 persons were excluded from the sensitivity analyses.*

*^d^Due to missing covariate data in CCC2000, ≤5 persons were excluded in the adjusted analyses.*

*^e^Interpretation (adjusted analysis comparing participants from the CCC2000 total sample to participants from AHEAD): if two participants from CCC2000 or AHEAD with the same sex, age and BMI are compared with each other, the participant from AHEAD will have a 16% (95%CI: 0%, 31%) lower median HCC than the participant from CCC2000.*

*^f^Interpretation (adjusted analysis comparing participants from the CCC2000 subgroup to participants from AHEAD): if two participants from the CCC2000 subgroup or AHEAD with the same sex, age and BMI are compared with each other, the participant from AHEAD will have a 15% (95%CI: -8%, 33%) lower median HCC than the participant from the CCC2000 subgroup.*

Supplementary Table 3 (ST3)

#### Crude and adjusted association between hair cortisol concentration and self-perceived stress in AHEAD using multiple linear regression

|  | **Dependent variable: PSS total score** | | |
| --- | --- | --- | --- |
| **Model^a^** | **β** | **95% CI** | **P** |
| **Crude (n=91)** | |  |  |
| - HCC spline 1 | -2.14 | -13.18; 8.89 | 0.700 |
| - HCC spline 2 | 64.35 | -145.22; 273.92 | 0.543 |
| - HCC spline 3 | -180.34 | -670.03; 309.34 | 0.466 |
| - HCC spline 4 | 163.01 | -187.13; 513.14 | 0.357 |
| **Adjusted for sex, age and BMI (n=91)** | |  |  |
| - HCC spline 1 | -2.34 | -13.73; 9.05 | 0.684 |
| - HCC spline 2 | 70.08 | -147.74; 287.91 | 0.524 |
| - HCC spline 3 | -194.19 | -703.75; 315.37 | 0.451 |
| - HCC spline 4 | 173.12 | -191.56; 537.79 | 0.348 |

*BMI: Body Mass Index, CI: confidence interval, HCC: hair cortisol concentration, n: number in specific population, PSS: Perceived Stress Scale, ref.: reference group*

*^a^HCC was modelled using restricted cubic splines.*
